# *De novo* priming: driver of immunotherapy responses or epiphenomenon?

**DOI:** 10.1042/EBC20220244

**Published:** 2023-09-28

**Authors:** Alexander L. Young, Tara Lorimer, Sarwah K. Al-Khalidi, Edward W. Roberts

**Affiliations:** 1 CRUK Beatson Institute, Glasgow, U.K.; 2 School of Cancer Sciences, University of Glasgow, Scotland, U.K.

**Keywords:** cancer, immunology, T-cells

## Abstract

The introduction of immunotherapy, in particular immune checkpoint inhibition, has revolutionised the treatment of a range of tumours; however, only a minority of patients respond to these therapies. Understanding the mechanisms by which different immune checkpoint inhibitors work will be critical for both predicting patients who will respond and to developing rational combination therapies to extend these benefits further. The initiation and maintenance of anti-tumour T cell responses is a complicated process split between both the tumour microenvironment and the tumour draining lymph node. As understanding of this process has increased, it has become apparent that immune checkpoint inhibitors can act both within the tumour and in the draining lymph node and that they can target both already activated T cells as well as stimulating the priming of novel T cell clones. Currently, it seems likely that immune checkpoint inhibition acts both within the tumour and in the tumour draining lymph node both reinvigorating existing clones and driving further *de novo* priming of novel clones. The relative contributions of these sites and targets may depend on the type of model being used and the timeline of the response. Shorter models emphasise the effect of reinvigoration in the absence of recruitment of new clones but studies spanning longer time periods examining T cell clones in patients demonstrate clonal replacement. Ultimately, further work is needed to determine which of the diverse effects of immune checkpoint inhibitors are the fundamental drivers of anti-tumour responses in patients.

## Introduction

The initial groundwork establishing the immune system as having a potential role in cancer therapy can be traced back as early as the late 1800s German physicians, when Busch and Fehleisen noted an epidemiological association between erysipelas infections and spontaneous tumour regression [1,2]. Later work by William Coley extended these findings by deliberately causing erysipelas infections to treat tumours. This work ultimately led him to conduct trials administering bacterial products therapeutically producing striking results [3]. A lack of mechanistic understanding, as well as the advent of surgery and radiotherapy prevented the wider adoption of these methods at the time. Independently of these studies in infection biology, early immunologists were investigating how the immune system may interact with cancer. In 1909 Ehrlich hypothesised that the immune system continuously eliminated nascent malignancies [4] and then in 1943, Ludwig Gross demonstrated that mice which had tumours surgically resected were protected against subsequent re-challenge establishing that the immune system could mount protective immune responses against tumours [5]. From these two lines of investigation there have long been indications that the immune system had potential to treat malignancy, but outgrowth of clinically relevant tumours indicated that tumours developed mechanisms by which to evade this immune control. Indeed, recently it has become clear that the immune system plays various roles in shaping tumour development and some of these may account for this failure of control; for example, a series of experiments carried out in the Schreiber lab demonstrated that methylcholanthrene-induced tumours developing in the presence of an adaptive immune system are less immunogenic and more readily accepted upon transplantation into immunocompetent recipient mice [6]. With these more recent findings it was unclear to what extent immunotherapy would be a viable approach to treating tumours, but when in 2011 US regulators approved Ipilimumab, an immune checkpoint inhibitor (ICI) targeting CTLA-4, for the treatment of advanced metastatic melanoma, they were signalling the start of a previously stalled era of cancer immunotherapy. In the years since, approaches from adoptive T cell transfer, chimeric antigen receptor T cell therapy, adjuvant administration and vaccination approaches have been taken to improve anti-tumour immune responses as reviewed recently [7]. However, despite these diverse approaches, the most successful approach in solid tumours remains ICI, where antibodies against key regulators of T cell function block inhibitory signals leading to improved T cell quality and quantity [8,9]. Given their central importance understanding the mechanism of action of these ICI is of central importance in delivering the full benefits of immunotherapy.

## Approaches to immune checkpoint inhibition

As stated previously, the first ICI approved by US regulators targeted CTLA-4 which was first described as a negative regulator of T cell function [10]. By competing with CD28 for binding of the costimulatory molecules CD80/86 expressed by antigen presenting cells, CTLA-4 was shown to reduce T cell activation and blockade of this receptor led to increased T cell activation and, critically, tumour regression in a mouse model of melanoma [11]. This signalled that reinvigoration to T cell function represented a potential avenue for immunotherapy. Indeed, other immune checkpoints have subsequently been identified and inhibitors generated to similarly boost anti-tumour T cells [12]. The most well described and widely used of these is represented by the PD-1/PD-L1 axis, which has been targeted with blocking antibodies against both the receptor and its ligand [13]. Inhibitors of this pathway are now approved for the treatment of, amongst others, melanoma, non-small cell lung carcinoma, renal cell carcinoma, bladder carcinoma and hepatocellular carcinoma, with immune checkpoint blockade representing first line treatment in several of these indications (Table 1)[8,14]. Despite the success of these ICI in the treatment of a range of tumour types, they have shown efficacy in only a subset of patients, with approximately 20–50% of patients responding in some indications, leaving a majority of patients receiving no clinical benefit from these treatments [15,16]. There remain two broad major challenges for ICI therapies: how to determine which patients will respond to treatment, and how to extend these benefits to further patients. The first of these questions has been the subject of a diverse literature seeking biomarkers of patients likely to respond to therapy from the abundance of PD-L1 expression in the tumour microenvironment to predict responsiveness to ICI targeting the PD-1:PD-L1 axis [17,18], through to evaluations of the immune composition of the tumour microenvironment [19]. This attempt to identify biomarkers has been extensively reviewed recently and so will not be further addressed [20]. The second question, that of how to extend the range of patients receiving benefit from ICI, has been approached in a variety of ways largely characterised by either the development of ICIs targeting novel immune checkpoints (Table 2) [7] or through the development of combination therapies. The latter of these approaches have led to increased responses although also increased occurrences of immune related adverse effects [7,16]. These attempts at rational combination therapies rely on an understanding of the fundamental mechanism of action of these ICI and there has been significant progress in this over the decade since Ipilimumab was first approved.

**Table 1:** Summary of approved immune checkpoint inhibitors for the treatment of various cancers

Approved agents	Indications	Line of treatment			Clinical trial
		1st	2nd		
***PD-1 inhibitors***					
Pembrolizumab	Melanoma	X	X		KEYNOTE Trials
	NSCLC	X	X		
	UC		X		
	cHL		*X	*following failed autologous stem cell transplant (ASCT)	
	HNSCC	X	X		
	RCC	*X	X	*with axitinib or with lenvatinib	
	MSI-H or dMMR	X	X		
	OC	*X		*with platinum + fluoropyrimidine-based chemotherapy	
	TNBC	*X	*X	* with chemotherapy	
	EC		*X	* with lenvatinib	
	Cervical	*X	*X	* with chemotherapy +/- bevacizumab	
Nivolumab	Melanoma	X	X		CheckMate Trials
	NSCLC	*X	X	*with ipilimumab + platinum-based chemotherapy for tumours with no sensitising EGFR mutation or ALK translocation	
	RCC	*X	X	*with ipilimumab or cabozantinib	
	HNSCC		X		
	cHL		X		
	MPM	*X		* with ipilimumab	
	UC		X		
	dMMR or MSI-H CRC		*X	* with ipilimumab	
	OSCC	*X	X	*with ipilimumab or fluoropyrimidine- + platinum-based combination chemotherapy	
	Gastric, GEJ, or oesophageal adenocarcinoma	*X		*with fluoropyrimidine- + platinum-based combination chemotherapy	
Cemiplimab	NSCLC	X			Libtayo Trials
	BCC		X		
	CSCC		X		
***PD-L1 inhibitors***					
Atezolizumab	NSCLC	X	X		IMvigor and IMpassion
	UC		X		
	SCLC	*X		*with carboplatin + etoposide	
	TNBC	*X		*with nab-paclitaxel	
	HCC	*X		*with bevacizumab	
Avelumab	MCC	X	X		Javelin trials
	UC	*X		*maintenance post platinum-based chemotherapy	
	RCC	X			
Durvalumab	NSCLC		X		Pacific and Study 1108 trials
	SCLC	*X		*with etoposide + either carboplatin or cisplatin	
	BTC			*with gemcitabine and cisplatin	
***CTLA-4 inhibitors***					
Ipilimumab	Melanoma	X	X		EORTC 18071 trial
	RCC	*X		*with nivolumab	CheckMate Trials
	NSCLC	*X		*with nivolumab + platinum-based chemotherapy	
	MPM	*X		*with nivolumab	
	dMMR or MSI-H CRC		*X	*with nivolumab	
	OSCC	*X		*with nivolumab	

Abbreviations: BCC, basal cell carcinoma; BTC, biliary tract cancer; cHL, classical Hodgkin lymphoma; CRC, colorectal cancer; CSCC, cutaneous squamous cell carcinoma; dMMR, deficient mismatch repair; EC, endometrial carcinoma; GEJ, gastro-oesophageal junction; HCC, hepatocellular carcinoma; HNSCC, head and neck squamous cell carcinoma; MCC, Merkel cell carcinoma; MPM, malignant pleural mesothelioma; MSI-H, high microsatellite instability; NSCLC, non-small cell lung cancer; OC, oesophageal carcinoma; OSCC, oesophageal squamous cell carcinoma; RCC, renal cell carcinoma; SCLC, small cell lung cancer; TNBC, triple-negative breast cancer; UC, urothelial carcinoma.

**Table 2:** Summary of novel immune checkpoints with the potential for therapeutic exploitation

Regulator	Potential mechanism
Lymphocyte activation gene 3 (LAG3)	Blocks CD4 contact sites on MHC class II proteins and reduces T-cell activation
T-cell immunoglobulin 3 (TIM3)	Induces effector T-cell death upon galectin-9 stimulation
V-domain immunoglobulin suppressor of T-cell activation (VISTA)	Inhibits T-cell proliferation and reduces T-cell cytokine production
B7-H3	Mechanism not fully understood but thought to play an inhibitory role in T cell proliferation
T-cell immunoreceptor with immunoglobulin and immunoreceptor tyrosine-based inhibitory motif domains (TIGIT)	Reduces effector T-cell proliferation by down-regulating the TCR-α chain and molecules that comprise the TCR complex

## Initiation and maintenance of anti-tumour T cell responses

Despite these advances, a major hindrance to solving these challenges lies in our relatively poor understanding of the initiation and maintenance of anti-tumour T cell responses. As in other settings, anti-tumour T cell responses are initiated when conventional dendritic cells (cDC) take up tumour antigens in the context of damage associated molecular patterns, mature and migrate to the tumour draining lymph node (tdLN) where they present antigen in the context of MHC to antigen specific T cells [21–23]. As in the initiation of anti-viral T cell responses, Batf3 and IRF8 dependent cDC1 have been shown to be critical in the initiation of anti-tumour CD8^+^ T cell responses [22,23] with cDC2 having a central role in driving the CD4+ T cell response [24]. This summary, however, oversimplifies the situation with considerable cross-talk existing between these cDC and T cell subsets as demonstrated by the fact that deletion of MHCII on XCR1+ cDC1 cells prevents initiation of anti-tumour CD4+ T cell responses and subsequent licensing of the cDC1 cells in a mouse model of melanoma [25]. There also exists evidence for the involvement of antigen transfer to tdLN cDC subsets which coordinate T cell help in a viral setting and that priming by these tdLN resident cDC cells is relatively poor in the tumour setting [26]. More recently, it has also been shown that in transplanted mouse models of melanoma and prostate cancer, CD8^+^ T cells primed in the tdLN acquired expression of the activation marker PD-1 while maintaining expression of the transcription factor Tcf7 and remaining negative for effector cytokines [27]. These T cells subsequently trafficked to the tumour microenvironment where they interact with cDC1 within the tumour microenvironment and gain full effector function [27]. Indeed some of these T cells appear to remain resident within dense clusters of antigen-presenting cells and maintain expression of Tcf7 even as they develop full effector function, representing a population of precursors of exhausted T cells (T_PEX_) [27]. In a viral setting it was shown that cDC1 were critical for the maintenance of the niches maintaining T_PEX_ and that T cells which exited these niches gradually became exhausted (T_EX_), losing expression of Tcf7 and, ultimately, effector molecules [28]. This loss of T cell effector functions within the tumour microenvironment (TME) is initially plastic but ultimately becomes irreversible due to changes in the epigenome, meaning T_EX_ remain exhausted even once removed from the TME [29,30]. Recent work from the Withers lab has added further complexity to the system. Using the Kaede mice to label intra-tumoural immune cells in a subcutaneous model of colon cancer, they showed that Tcf7+ T_PEX_ can reverse migrate back to the tdLN potentially seeding the tdLN-derived tumour-specific memory cells (T_SM_) population there [31]. These T_SM_ cells are marked by expression of PD-1 and Tcf7 [32], thus resembling the stem-like cells generated during the initial partial priming occurring in the tdLN [27]. All this complexity makes unpicking the site of action of ICIs challenging as different cDC subsets have varied roles in both the TME and the tdLN and diverse T cell subsets continuously migrate between the two sites with overlapping markers characterising their different states. Given the complexity of this process it is perhaps understandable that it is uncertain exactly where therapies such as αCTLA4 and αPD-1 exert their anti-tumour effects. This lack of clarity makes it more challenging to design future approaches to improve the efficacy of these therapies and so there is a pressing need to identify the cells upon which these drugs act. One key question which remains is to what extent *de novo* priming of new T cell clones is required to drive the anti-tumour effects of ICIs as compared to reinvigoration of already primed, existing anti-tumour T cells.

## Effects of immune checkpoint inhibitors in the tumour microenvironment

Given that both CTLA-4 and PD-1 are up-regulated upon T cell activation, early work centred around the roles of ICI reactivating already primed T cells predominantly within the TME [15]. CTLA-4 has been shown to act: T cell intrinsically by, for example, competing with CD28 for binding to costimulatory molecules [33]; by altering regulatory T cell (T_REG_) function [34,35] or by reducing the levels of cDC CD80/86 reducing their ability to further stimulate T cell activation [35,36]. PD-1 on the other hand, in response to binding its ligands PD-L1 or PD-L2 [37,38] recruits the phosphatase SHP-2 to its intracellular tail attenuating T cell receptor (TCR) signalling and reducing downstream effector production [39]. PD-L1 is up-regulated in response to IFNy signalling within the TME and can be present on both tumour cells and infiltrating immune populations [40]. Thus PD-1:PD-L1 signalling is engaged upon the initiation of an effector response, acting as a bona fide checkpoint that dampens further damage to tissue in the absence of continued strong signalling through the TCR. There are also secondary effects of activating T cells with ICI within the TME. Increased production of IFNγ by T cells downstream of ICI treatment leads to increased activation of intra-tumoural cDC and their subsequent support of T cell function through production of IL-12 [41]. Interestingly this production of IFNγ likely also induces up-regulation of PD-L1 on cDC [42] and other studies have shown that cDC are the critical source of PD-L1 in the TME in predicting responsiveness to ICI in some mouse models of cancer [42,43]. This suggests that the character of cDC and their relative responsiveness to IFNγ is likely of critical importance in determining responses to ICI. Indeed, other recent studies have shown that upon ICI the stem-like T_PEX_ population residing within the TME rapidly expand and regenerate T_EX_ cells leading to tumour control [44]. As stated previously these T_PEX_ reside in niches rich in cDC and so these effects on cDC likely impact T_PEX_ disproportionately. The centrality of these cells in responses to ICI has been highlighted by a recent study where ICI was combined with FTY-720 to eliminate the contribution of the tdLN to the therapeutic response. In the absence of migration from the tdLN there was equivalent control of B16 tumours over a period of 6 days and this required the presence of Tcf7+ T_PEX_ within the TME [44]. This demonstrates the potential for reinvigoration of a subset of T cells within the TME which can lead to tumour control, a conclusion supported by other studies showing the proliferation of these T_PEX_ within the TME of a range of tumours in response to ICI [45]. While these data may seem to suggest that TME resident T_PEX_ are centrally important to responses to ICI there is a significant caveat that by using rapidly growing subcutaneous models, responses are monitored over a brief time-span and so the question remains about whether these intra-tumoural T_PEX_ cells are sufficient to maintain anti-tumour responses over the longer time periods associated with treatment of patients. Despite this, the importance of TME resident T_PEX_ cells in human cancer can be highlighted by the fact that the abundance of Tcf7+ CD8+ T cells within the TME of melanoma patients is associated with responsiveness to ICI [46]. Interestingly, given their central role in maintaining a T_PEX_ niche within the TME, it has also been shown that the abundance of cDC1 also correlates with responsiveness to αPD-1 therapy in both melanoma and head and neck squamous cell carcinoma [19]. Thus, the composition of the TME, particularly in regards to the abundance of T_PEX_ appears to be associated with more positive outcomes. The ability to deduce the site of action of ICI in these human studies is, however, limited due to the absence of matched samples from the tdLN and the reasonable hypothesis that T_PEX_ abundance in the TME may correlate with the environment in the tdLN given that T_PEX_ both originate from precursors within the tdLN and themselves act as a source for T_SM_ [27,31].

## Evidence for the importance of the tumour draining lymph node

Despite these studies showing the centrality of tumour resident T cells for tumour control, there is also conflicting evidence for the importance of the tdLN in responses to ICI [47]. Conceptually, given that systemic administration of ICIs will likely lead to their presence in the tdLN this site could be implicated in two main ways: it could be either an important site for downstream effects of inhibition occurring in the TME or it may represent the site of ICI activity itself. The first of these models is illustrated by a study where a CTLA-4 antibody was used to deplete T_REG_ specifically within the TME in a model of melanoma [24]. In this study, depletion of T_REG_ cells led to increased activation of cDC2 which migrated to the tdLN and subsequently improved anti-tumour CD4+ T cell responses. This priming in the tdLN was critical for the therapeutic benefit as inhibition of T cell egress from the lymph node with FTY-720 was sufficient to prevent the tumour regression [24]. In fact multiple groups have shown in pre-clinical models that the lymph node is also central for the function of αPD-1 therapies with surgical excision of the tdLN prior to therapy resulting in blunted therapeutic effects [48]. While these functions of the tdLN may be downstream of inhibition occurring in the TME, there is also evidence that ICI may directly exert an effect within the tdLN itself. Firstly, while it was originally assumed that the kinetics of PD-1 up-regulation during T cell activation suggested activity would be restricted to the TME, it has now been shown that PD-1+ T cells arise early during expansion following priming [49] and indeed PD-1+ tumour specific T cells can be found in the tdLN [27,31,50]. Furthermore, these may be newly activated or T_SM_ which have migrated back from the TME to the tdLN [31]. These cells are high in PD-1 expression and so could represent targets for PD-1 blockade. Moreover, there is an abundance of PD-L1 expressing cells, including cDC2, which can act to restrain the functionality of these T cells within the tdLN [51]. Interestingly there may be further interplay between ligands involved in both of these pathways with recent work highlighting the ability of CD80 to interact with PD-L1 in cis blocking engagement with PD-1 and preventing trogocytosis of CD80 from cDC by CTLA4 [52,53]. As such both ICI approaches impact the ability of cDC in the tdLN to efficiently activate T cells and give some indication as to why such synergy has been observed. This raises a question about why in previously mentioned studies treatment with FTY-720 showed no impact on the efficacy of ICI treatment. One possible answer to this is presented by Dammeijer et al. who used a slower growing AC29 model of lung cancer and treated mice with αPD-L1 over an extended period. In this model, contrary to what was seen in the B16 model, FTY-720 administration abrogated the therapeutic efficacy of systemic ICI treatment [51]. Even more strikingly, delivery of greatly reduced amounts of ICI intrapleurally to target PD-1 specifically in the tdLN resulted in tumour control similar to that seen with systemic administration [51]. These PD-1:PD-L1 interactions seen in mouse tdLN have also been demonstrated to occur in the tdLN of patients with stage II melanoma and higher incidence of such interactions correlated with poorer survival post-surgical resection [51]. In fact direct delivery of ICIs to the tdLN has been shown to induce optimal responses in several studies [54–57] highlighting the critical role of the tdLN in driving the effects of ICI. Another potential reason for the discrepancies between the relative importance of the tdLN in responses to ICI in mouse models lies in the fact that many mouse models have limited lymphatic development and alteration of the tdLN. These tend to occur more in slower growing tumour models again highlighting the importance of the use of these models to better understand the impacts of ICI in patients [58]. Furthermore the tdLN itself may not be obvious with lymphatic co-option leading to drainage to nodes other than that expected due to the anatomical site of the tumour [59]. This can also be the case in patients where the tdLN is not always obvious complicating the types of analyses which may clarify these issues. Despite this, the increased understanding of the presence of T_SM_ within the tdLN raises the question of whether these interventions function through reinvigoration of existing responses or, in fact, drive *de novo* priming.

## *De novo* priming or reactivation?

**Figure 1:**
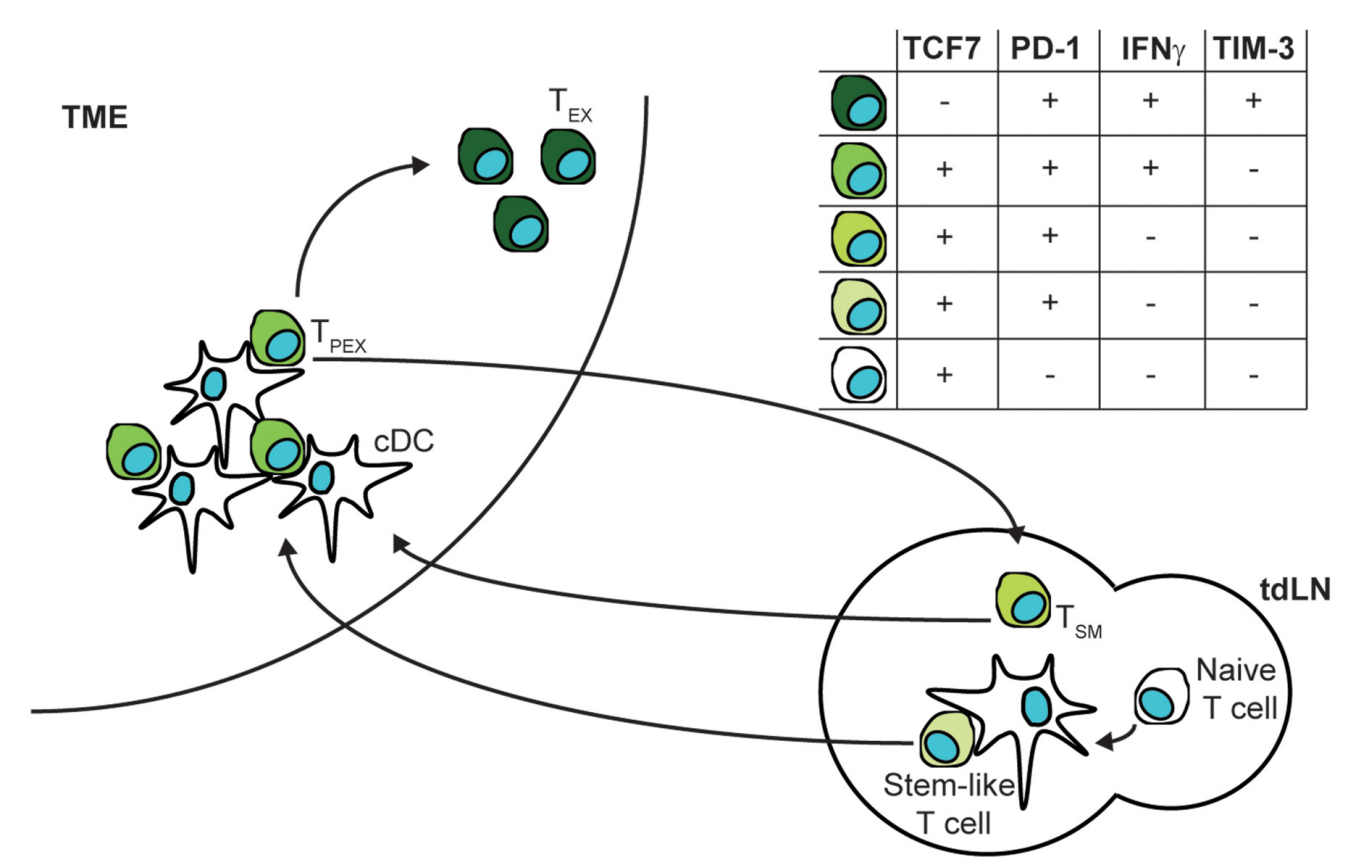
Relationships between T cell subsets in the TME and tdLN Naïve T cells are activated in the tdLN expressing PD-1 while maintaining expression of TCF7 in a partially activated, stem-like state. These stem-like T cells migrate to the TME inhabiting cDC rich niches where further stimulation results in acquisition of effector functions but maintenance of TCF7 expression. These T_PEX_ cells can reverse migrate back to the tdLN seeding a T_SM_ pool which retain TCF7 and PD-1 expression while losing effector functions or they can progress to differentiate into T_EX_, losing expression of TCF7 and gaining TIM-3 expression.

The difficulty of defining the contribution of *de novo* priming of new T cell clones as opposed to reinvigoration of existing responses arises since the tdLN contains both naïve tumour-specific T cells, partially primed T cells which may arise during priming due to a separation of activation and full acquisition of effector function and T_SM_ cells which may be seeded by T cells returning to the tdLN from the TME (Figure 1). As such, while it has been shown that ICI treatment drives expansion of T cell clones outside of the TME which acted to replenish TME T cells over time, the source and character of the cells giving rise to this expansion is unclear [60]. The systemic response to ICI has also been shown to be of critical importance in several mouse models [61], however, even in this more manipulable setting it was not clear which subsets of T cells may be critical for the observed responses. One approach to unpick this was taken by Huang et al. where they compared the capacity of T_SM_, T_PEX_ and T_EX_ cells to induce tumour control in a mouse model of melanoma showing that transfer of T_SM_ led to the best tumour control with T_PEX_ showing intermediate control and T_EX_ showing the least. When mice were subsequently treated with αPD-L1, T_SM_ were also shown to expand more than did transferred T_PEX_ cells [32]. Critically, transfer of T_SM_ was sufficient to restore the effect of αPD-L1 therapy in mice post-surgical excision of their tdLN suggesting that the tdLN may simply acting as a store of T_SM_ cells and that ICI works by altering their interactions with cDC within the node [32]. This study, however, again suffers from the fact that it occurs in a fast-growing tumour model which necessitates responses being monitored over the course of less than a week. Supporting this conclusion was work in the autochthonous KP-NINJA mouse model, which is much slower to develop. Connolly et al. demonstrated that the TCR repertoire in the tdLN and the TME remained closely related throughout tumour development suggesting a continued relationship between these two pools [50]. Crucially treating these mice with FTY-720 for three weeks led to a loss of TME T cells but had no effect on the numbers in the tdLN, suggesting that the tdLN acts as a source of T cells throughout tumour development [50]. The T cells in the tdLN had a stem-like phenotype distinct to naïve T cells suggesting that under normal conditions the ongoing tumour response may be maintained by existing clones and so this could represent contributions from T_SM_ or partially primed, stem-like T cells. This study did not, however, address what occurs during ICI. As such there is still some doubt as to whether naïve T cells may be *de novo* primed in response to ICI and whether this is important for responsiveness. To get at this there have been several studies which have intriguingly suggested that *de novo* priming may have a role in the response to ICI in patients. In 2018, 21 patients with untreated, surgically resectable non-small cell lung carcinoma (NSCLC) were administered Nivolumab every 2 weeks and their tumours were removed 4 weeks after the initiation of treatment. Peripheral blood was analysed and T cell clones reactive against candidate neo-antigens prior to treatment were identified. Subsequently to treatment this process was repeated and several new T cell clones were found to have expanded which reacted to candidate neo-antigens; these new T cell clones were not identified in the pre-treatment samples [62]. This indicated that new T cell clones were being stimulated in response to ICI. Later analyses performing single cell RNAseq and paired TCR sequencing on samples from patients with basal or squamous cell carcinoma demonstrated that there was an expansion of novel TCR clonotypes not previously found in the TME following PD-1 blockade [63]. Given the interconnectedness of the TME and the tdLN it appears unlikely that these represented existing T_SM_ cells and were instead likely derived from naïve T cells undergoing *de novo* priming. This appears a reasonable conclusion, especially given previous data showing that treatment with αPD-1 improves cDC ability to prime in the tdLN in mouse models. Despite these findings, it is unclear as to how important these newly primed T cells are in generating the anti-tumour response post ICI treatment. Given the ability for tumour clones to evolve and lose expression of neo-antigens, the generation of new T cell responses post ICI treatment is an attractive prospect. However, despite these tantalising patient data it is unclear how important these new responses are in driving ICI responsiveness. A further consideration is that both the PD-1 and CTLA-4 pathways are important in the maintenance of peripheral tolerance through a variety of mechanisms in both peripheral tissues and within the lymph node [64–66]. As such the use of ICIs has been associated with a number of immune related adverse events (iRAEs) leading to reduced use of CTLA-4 based therapies in many areas [67]. These iRAEs and indeed the delayed iRAEs observed with PD-1 based therapies, may also be caused by *de novo* priming of self antigen specific T cell clones [68]. This represents another key area to investigate moving forward but is outside the scope of this review.

## Conclusion

In conclusion, due to the complex nature of the anti-tumour T cell response, it is unclear the relative contribution of rejuvenation of tumour infiltrating T cells, be they T_EX_ or T_PEX_, as compared with that of tdLN-derived T cells. Furthermore questions remain about the relative contribution of reactivation of T_SM_ as compared to *de novo* priming of naïve T cells. As noted, many of these questions remain due to the difficulties inherent in unpicking a complex interconnected system represented by the wider tumour macroenvironment incorporating both the TME and the tdLN; however, other questions arise from the necessity to better understand and position the mouse models being used for such studies. A pertinent example of this comes from the different conclusions reached about the impact of FTY-720 treatment on ICI outcomes. In some mouse models FTY-720 leads to no alteration in responsiveness to ICI whilst in others it completely abrogated any response. These questions are not trivial as understanding better the mechanism of action of ICI will inform better combination therapeutic approaches moving forward. As such, there is a pressing need to better characterise existing tumour models and to develop new ones which may address outstanding questions. Despite these caveats, it appears likely that in patients at least there is a contribution made to the ongoing T cell response from *de novo* priming during ICI therapy, however, whether that is the main driver or merely a passenger in the effective response remains to be seen.

## Summary

- New approaches are needed to increase the number of patients benefitting from ICI therapy and this requires greater understanding of their mechanism of action.
- Different model systems provide data suggesting roles for ICI both within the TME and in the tdLN.
- Data from patients treated with ICI suggest clonal replacement of T cells indicating *de novo* priming of new T cell clones although the necessity of this for responsiveness has not been elucidated.

## Open Access

Open access for this article was enabled by the participation of the University of Glasgow in an all-inclusive *Read & Publish* agreement with Portland Press and the Biochemical Society under a transformative agreement with JISC.

## Abbreviations

cDC: conventional dendritic cell
ICI: immune checkpoint inhibitors
iRAE: immune related adverse event
NSCLC: non-small cell lung cancer
TCR: T cell receptor
tdLN: tumour draining lymph node
T_EX_: exhausted T cells
TME: tumour microenvironment
T_PEX_: precursors of exhausted T cells
T_REG_: regulatory T cells
T_SM_: tumour specific memory T cells
